# “Restoring That Faith in My Shoulder”: A Qualitative Investigation of How and Why Exercise Therapy Influenced the Clinical Outcomes of Individuals With Rotator Cuff–Related Shoulder Pain

**DOI:** 10.1093/ptj/pzad088

**Published:** 2023-07-13

**Authors:** Jared K Powell, Nathalia Costa, Ben Schram, Wayne Hing, Jeremy Lewis

**Affiliations:** Faculty of Health Science and Medicine, Bond Institute of Health and Sport, Bond University, Robina, Australia; Faculty of Medicine and Health, Sydney School of Health Sciences, The University of Sydney, Sydney, Australia; Faculty of Health Science and Medicine, Bond Institute of Health and Sport, Bond University, Robina, Australia; Faculty of Health Science and Medicine, Bond Institute of Health and Sport, Bond University, Robina, Australia; Therapy Department, Central London Community Healthcare National Health Service Trust, London, United Kingdom; Musculoskeletal Research, Clinical Therapies, University of Limerick, Limerick, Ireland

**Keywords:** Exercise, Rotator Cuff, Shoulder, Shoulder Pain, Subacromial Impingement Syndrome

## Abstract

**Objective:**

Rotator cuff–related shoulder pain (RCRSP) is the most common form of shoulder pain. Exercise therapy is a first-line recommended treatment for RCRSP. However, the causal mechanisms underpinning the benefits of exercise for RCRSP are not well understood. Moreover, how individuals with lived experience of RCRSP believe exercise helped or did not help them is unknown. This study aimed to gain insights into how individuals with RCRSP believe exercise influenced their shoulder pain and identify the clinical conditions that promoted or inhibited their beliefs.

**Methods:**

This qualitative study was underpinned by a critical realist approach to thematic analysis. Participants were recruited using hybrid purposive and convenience sampling techniques. Each participant attended an online semi-structured interview. The data were coded by 2 members of the research team (J.K.P. and N.C.) and verified by a third (B.S.). Recruitment continued until theoretical sufficiency was achieved. Participants reviewed and validated preliminary causal explanations.

**Results:**

Three causal explanations were consistently expressed by 11 participants to explain the benefits of exercise therapy: (1) shoulder strength; (2) changes to psychoemotional status; and (3) exercise has widespread health effects. However, the activation of these causal mechanisms depended on (1) the presence of a strong therapeutic relationship; (2) the provision of a structured and tailored exercise program; and (3) experiencing timely clinical progress.

**Conclusion:**

Participants believed exercise improved their shoulder pain through associated health benefits, improved shoulder strength, and psychoemotional variables. Whether an exercise program was able to cause a clinical improvement for an individual with RCRSP was contingent on clinical contextual features. Thus, the clinical context that an exercise program is delivered within may be just as important as the exercise program itself.

**Impact:**

Exercise is a recommended first-line intervention to manage RCRSP. The results of this study suggest that a positive experience and outcome with exercise for RCRSP is contingent on several clinical contextual features, such as a strong therapeutic relationship. The clinical context that an exercise program is prescribed and delivered within should be considered by clinicians.

## Introduction

Shoulder pain is a common and often disabling musculoskeletal pain condition.^1^^,^^2^ In those afflicted, it impacts activities of daily living, sleep, physical and emotional function, and participation in valued activities.^2^^,^^3^ Rotator cuff–related shoulder pain (RCRSP) is a clinical term introduced in 2016,^4^ and it structurally refers to the muscles, tendons, and surrounding structures, such as bursae, bone, ligament, capsule, nerve, and their concomitant vascular tissues. RCRSP is the most common musculoskeletal shoulder condition and presents as shoulder pain and weakness, most commonly during shoulder elevation and external rotation.^1^^,^^4^ RCRSP was introduced to avoid problematic pathoanatomical diagnoses such as subacromial impingement syndrome and non-traumatic rotator cuff tears. Internationally, the term RCRSP is preferred over subacromial pain syndrome,^5^ which refers to an anatomical location that is difficult to comprehend.

Both exercise therapy and education are recommended as the principal non-surgical approach for managing RCRSP.^6^ This is reflected in evidence–based clinical practice guidelines, where exercise therapy is the only treatment featured in all hitherto guidelines for RCRSP.^7^^,^^8^ Exercise therapy results in comparable outcomes to more invasive treatment options, such as rotator cuff repair^9^ and subacromial decompression surgery,^10^ corticosteroid,^11^ and platelet-rich plasma injections.^12^ However, it must be acknowledged that exercise is far from a panacea for RCRSP.^13^ Ultimately, it is the clinician and patient, together in partnership, who are tasked with devising an agreed treatment plan, which may include an exercise program.

There exists modest evidential support for exercise therapy to manage RCRSP^14–16^; however, despite a high volume of research testing various combinations of exercise programs, the causal mechanisms underpinning the effect of exercise are largely unknown. Moreover, the clinical conditions (context) that promote or inhibit possible causal mechanisms have received inadequate attention.

Previous qualitative research endeavors exploring RCRSP have focused on the impact of diagnostic labels,^17^ patient beliefs about the cause of their shoulder pain,^18^ general experiences with exercise,^19^^,^^20^ barriers and enablers to exercise,^21^ and the biopsychosocial impact of living with RCRSP.^22^ An in-depth exploration of the causal explanations expressed by individuals with lived experience of RCRSP about how exercise may have helped them, or not, is missing. Furthermore, knowledge is lacking about the clinical conditions that could trigger or hinder the activation of said causal mechanisms. Acquiring first-person perspectives about causal explanations of exercise may help clinicians understand how an individual with RCRSP might comprehend the utility of an exercise program and identify the clinical conditions that could maximize the benefits of an exercise regimen. Commonly proposed causal explanations could subsequently be tested in quantitative research, using mediation analysis, for example. As such, this study aimed to gain insights into how individuals with RCRSP believed exercise helped their shoulder pain and identify the clinical conditions that promoted or inhibited this.

## Methods

### Design

A qualitative study using in-depth semi-structured interviews conducted in accordance with the Standards for Reporting Qualitative Research.^23^

### Qualitative Approach and Research Paradigm

This qualitative research investigation was underpinned by a critical realist philosophy.^24^ Critical realism is a philosophy of science that integrates a realist ontology, which posits there is something real in the world to find out about, with a relativist epistemology, which is based on the premise that individuals will come to know different things in different ways.^25^^,^^26^ Using a critical realist approach to thematic analysis is thought necessary when the primary research question seeks a causal explanation to a particular event or experience.^27^ The purpose of a critical realist approach to thematic analysis is to produce the most plausible explanations of the mechanisms that cause events or experiences. A critical realist approach to thematic analysis follows a 5-step process, where it is possible, and encouraged, to move freely back and forth between the steps.^27^ These steps are explicated in detail in the following sections.

### Researcher Characteristics and Reflexivity

Lead investigator (J.K.P.) is an Australian trained physical therapist and PhD candidate with 12 years of clinical experience, who has a particular interest in the assessment and management of musculoskeletal shoulder disorders and who has received training in qualitative research. N.C. is a musculoskeletal researcher who has a PhD in physical therapy and draws from both social science and public health lenses when conducting qualitative research. Fellow investigators (B.S., W.H., and J.L.) are experienced researchers and musculoskeletal clinicians with extensive experience in both quantitative and qualitative research methods, each with more than 100 scientific publications. We recognize that the results presented here are informed by our multiple and varied positionalities.

### Data Collection Methods

The lead investigator (J.K.P.) conducted 7/11 interviews and N.C. conducted 4/11 interviews. All but 1 interview was conducted via Zoom video conferencing software. The remaining interview was conducted over the telephone using audio recording only as the participant did not like using Zoom. Investigators and participants engaged in the interviews predominately from a home setting, and on some occasions from their work setting. In all cases, interviews were conducted 1 to 1 with a single investigator. Video and audio interviews were chosen to limit the constraints of geographic location and because this is often the preferred method of participants.^28^ Data collection commenced on August 2, 2022 and ended on December 5, 2022.

### Sampling Strategy

Participants were eligible to participate in this study if they met the selection criteria outlined in Table 1.

**Table 1:** Selection Criteria

Inclusion Criteria	Exclusion Criteria
Aged 18 and above	Had previous shoulder surgery on the affected arm within 12 mo of diagnosis of rotator cuff–related shoulder pain
Has had an episode of shoulder pain lasting more than 1 mo	Has a known inflammatory disorder (such as rheumatoid arthritis)
Has been assessed by a registered health care professional and been given a diagnosis of rotator cuff–related shoulder pain, which includes rotator cuff tear (full or partial thickness), rotator cuff tendonitis/tendinopathy/tendinosis, shoulder impingement syndrome, and subacromial bursitis	Had a fracture on the affected arm within 12 mo of diagnosis of rotator cuff–related shoulder pain
Has engaged with a rehabilitative exercise program as prescribed by a registered health care professional	Had been diagnosed with frozen shoulder within 12 mo of diagnosis of rotator cuff–related shoulder pain
Has proficiency in written and spoken English	Had known glenohumeral joint osteoarthritis at the time of diagnosis of rotator cuff–related shoulder pain
	Had displayed evidence of calcific tendinopathy within 12 mo of diagnosis of rotator cuff–related shoulder pain
	Had a history of shoulder dislocations or subluxations on the affected shoulder
	Had displayed signs and symptoms of cervicogenic shoulder pain and cervical radiculopathy within 12 mo of diagnosis of rotator cuff–related shoulder pain
	Had any condition of the arm/shoulder/nervous system that restricts being able to exercise it

Convenience sampling was used to recruit participants via the social media platforms Instagram, Facebook, and Twitter, and who presented to the lead investigators (J.K.P.) clinical practice. As some participants were known to the principal investigator as former patients, a co-investigator (N.C.) interviewed them. Purposive sampling was used to facilitate an even representation of sex and diversity in terms of duration of pain and occupation. As a research group, we acknowledge the ongoing debate regarding when to stop recruitment, as problems have been identified with the traditional “thematic saturation” concept.^29^^,^^30^ We opted for theoretical sufficiency as an alternative approach to ceasing data collection. Such an approach emphasizes how meaningful the data appears, with the view that if it adds no new perspectives and understandings to the topic explored, it is reasonable to infer that an appropriate sample size has been achieved.^30^^,^^31^

### Ethics

Ethical approval for this research project (project number JP03070) was obtained by the Bond University Human Research Ethics Committee. All participants provided written informed consent prior to interview.

### Pilot Interview

A pilot interview was recorded with an individual with a history of RCRSP, which lasted for 29 minutes. The pilot interview led to the refinement of language and structure of questions in the interview guide.

### Data Processing and Analysis

The interview audio files were transcribed verbatim using the digital platform Rev. Transcripts (Austin, TX, USA) were checked for accuracy against the original audio file, anonymized using pseudonyms and deidentified of relevant geographical locations, and subsequently uploaded to the electronic data management software, NVivo (Denver, CO, USA), for commencement of data analysis. For data analysis, we followed the 5-step critical realist approach to thematic analysis as described by Fryer.^27^ Investigators J.K.P. and N.C. independently read the first 3 transcripts with the research question in mind, and then met to develop preliminary codes. N.C. then coded 3 of the transcripts and J.K.P. coded 8. J.K.P. developed the causal explanations captured in our codes,^27^ which were discussed with the research team. The development of causal explanations employs retroductive reasoning, which allows for inferences to be made about what best explains a particular event or experience.^25^

### Techniques to Enhance Trustworthiness

We took several steps to enhance the trustworthiness and rigor of this qualitative research investigation. First, we had multiple interviewers (J.K.P. and N.C.) and coders (J.K.P. and N.C.). We then had an additional investigator (B.S.) review and verify these codes against the original transcripts. One investigator (J.K.P.) developed and refined the causal explanations in constant consultation with the research team. To enhance the credibility of data analysis and participant involvement, refined causal explanations were sent to study participants for an opportunity to provide feedback or request a modification.^30^^,^^32^

## Results

Twenty participants volunteered for this study, with 9 participants unable to meet the selection criteria (8 did not fulfill the clinical presentation of RCRSP, and 1 did not perform an exercise program). Eleven participants with RCRSP with a mean age of 36.5 years and median symptom duration of 16 months were enrolled in the study. The participants were from Australia (7), Canada (1), the United States (1), Germany (1), and South Africa (1). Further participant details are provided in Table 2. The average interview duration was 37 minutes (range 27–45 minutes). A decision was made to cease data collection when the final 3 interviews did not modify existing coding categories (theoretical sufficiency), and the data hitherto obtained were rich in depth and judged to add no new perspectives to the topic investigated. All participants were given the opportunity to review preliminary findings. Participants were satisfied that their individual experiences and perspectives were captured in the results of this study. Overall, exercise therapy was perceived to be a beneficial treatment for RCRSP, under certain conditions:

**Table 2:** Participant Information

Participant (Pseudonym) Sex, Age (y)	Occupation	Nationality	Duration of Symptoms	Diagnosis	Health Care Professional Consulted	Treatments Trialed	Imaging Received
Luke Male, 32	Maintenance professional	Australian	10 mo (ongoing)	Rotator cuff tendinopathy, subacromial bursitis, and shoulder impingement syndrome	General Practitioner Physical therapist	Rest and exercise–based rehabilitation	X-ray and ultrasound
Kevin Male, 31	Business owner	Australian	18 mo (ongoing)	Partial thickness rotator cuff tear and subacromial bursitis	Physical therapist	Rest, dry needling, and exercise–based rehabilitation	MRI
Claire Female, 36	School teacher	Australian	60 mo (ongoing)	Rotator cuff tendinopathy, partial thickness tear and subacromial bursitis	Osteopathy General Practitioner Physical therapist	Massage, dry needling, rest, ice, and heat pack, NSAIDs, and exercise–based rehabilitation	X-ray and ultrasound
Bill Male, 31	Electrical engineer	Australian	36 mo (recovered)	Shoulder impingement syndrome and subacromial bursitis	General Practitioner Physical therapist Massage therapist Chiropractor Exercise physiologist	NSAIDs, massage, dry needling, chiropractic adjustments, and exercise–based rehabilitation	X-ray, ultrasound, and MRI
Max Male, 52	General manager	Australian	9 mo (ongoing)	Rotator cuff tear and subacromial bursitis	General Practitioner Physical therapist Osteopath	Ice, massage, and exercise–based rehabilitation	X-ray and ultrasound
Jessica Female, 25	Student	South African	16 mo (ongoing)	Rotator cuff tendinopathy and subacromial bursitis	Orthopedic surgeon Physical therapist Biokineticist	Manual therapy, exercise–based rehabilitation, and NSAIDs	MRI
Matthew Male, 33	Strength and conditioning coach	Australian	24 mo (recovered)	Shoulder impingement syndrome	Physical therapist Chiropractor Exercise physiologist	Manual therapy, dry needling, chiropractic adjustments, and exercise–based rehabilitation	None
Ella Female, 65	Retired	American	5 mo (ongoing)	Shoulder impingement syndrome	Physician assistant Physical therapist	Exercise–based rehabilitation	X-ray
Charlotte Female, 26	Student	Canadian	132 mo (ongoing)	Rotator cuff tendinopathy	Physical therapist Massage therapist Chiropractor	Manual therapy, chiropractic adjustments, therapeutic ultrasound, and exercise–based rehabilitation	None
Lisa Female, 34	Nurse	Australian	6 mo (recovered)	Rotator cuff tear and subacromial bursitis	Orthopedic surgeon Physical therapist	Ice, analgesics, manual therapy, and exercise–based rehabilitation	MRI and ultrasound
John Male, 27	Personal trainer	German	4 mo (ongoing)	Subacromial bursitis and rotator cuff tear	Sports physician General Practitioner Physical therapist	Exercise–based rehabilitation, percussive therapy, cupping, massage therapy, NSAIDs, and topical heat cream	X-ray and ultrasound

### Condition 1: A Strong Therapeutic Relationship Is the Foundation for a Clinical Improvement With Exercise Therapy

All participants stressed their relationship with their treating clinician played an important role in their engagement in, and improvement through, exercise (Tab. 3). Based on participants’ responses, a strong therapeutic relationship built on trust, collaboration, reassurance, and empathy seemed to be the foundations for a positive experience with exercise. Conversely, a clinical interaction devoid of a strong therapeutic relationship, primarily because of poor communication or a lack of trust, meant that exercise could not contribute to clinical improvement in shoulder pain or function, either because the patient would seek a different therapist or would not do the exercises. Our interpretation from participants’ experiences is that the relational aspects of musculoskeletal health care can influence whether an exercise program is able to generate a positive clinical outcome (such as reduced shoulder pain) and highlights the importance of the patient–clinician relationship. Thus, we propose that the causal power of an exercise program to positively influence shoulder symptoms is conditional upon a strong therapeutic relationship between the clinician and patient.

**Table 3:** Supporting Quotes of Clinical Conditions That Promoted or Inhibited the Success of an Exercise Program for an Individual With Rotator Cuff–Related Shoulder Pain

Condition	Supporting Quotes
Condition 1: A strong therapeutic relationship is the foundation for a clinical improvement with exercise therapy	“*I think just with the relationship that I built with my physio and I knew that whatever he said I needed or didn’t need, and I trusted his advice, I trusted his education, and how we, I guess, just had that therapist and patient relationship. It was very, very comforting.*” (Charlotte)
	*“So I think that was really good too, because I got a much more trusting relationship [with the therapist], that they were just genuinely more interested in me trying to build long term strength and health rather than a repeat customer to some extent*.*”* (Bill)
	“*The way that it was communicated, it didn’t really fill me with confidence that there was a lot of buy-in to getting me out the other end, and I’d say as quickly as possible, because that my motivation was to get out the other end quickly*.” (Max)
	*“I never really liked the physio or the head of physio... He was very dismissive in general with a lot of conditions, especially anything to do with CrossFit. So when I went in and got seen, I didn’t get a lot of, I guess, interest. It was like, ‘Oh, another person with shoulder pain’ … And that’s when I went to the next physio.”* (Matthew)
	“*She could temper it in a positive way by telling me how far I’ve come. “That’s normal. You’re working those muscles, that’s normal. It’s got to be sore.” And so she always normalized everything for me. I’d always get really concerned I was making it worse*.”
	
Condition 2: The exercise program should be structured and tailored to the individual clinical presentation	*“And again, it seemed a little like it’s a generic document and stuff like that, so you sort of look at it and think, it’s just the same as lots of other people, whether it’s good or bad.”* (Max)
	*“Yeah, it felt like it was a more tailored approach to me about me getting better as opposed to just a cookie cutter; here’s a default program for six to eight weeks that should make you better. Everyone does the same thing approach”* (Bill)
	“*He guided me through not only with the exercises and giving me a trajectory, which was fantastic, because I really like planning and structure so what it’s going to look like in a month’s time, what it’s going to look like in two months’ time”* (Lisa)
	“*Probably relieved to have a bit of guidance, to have a plan. Because although I haven’t had any success, long term success with any of the treatments I’ve done, but just have a plan and someone to check in with fortnightly or monthly. So I felt like it was good and I’m held accountable*” (Claire)
	“*So if something felt too easy, I’m like, Ah, is it of benefit if it feels too easy, or if there’s pain there, is that an issue or not... But at the time, I still felt like I was on my own to figure it out, and he didn’t provide a follow-up appointment or anything. It was more just, “Yeah, do this for a couple weeks. Your shoulder will be fine. If it’s not fine, then we can chat later.” But there was no the check-ins scheduled or anything*.” (Kevin)
Condition 3: Timely clinical progress with exercise therapy matters	“*Okay, I can see the progress, I can see what’s happening with my shoulder.*” (Charlotte)
	*“What really helped absolutely in me not giving up, of course, is seeing progress.”* (John)
	“*It’s been four or five weeks, I’m already feeling an improvement in that now, I can demonstrate to you and I don’t have that same twinging, sharp impingement pain. And I put that down to the exercise that I’ve been doing religiously for the last five weeks. So that’s had a better outcome for me than the osteo treatments.* (Claire)
	“*But anyway, I like the exercises even though at first they made me very sore. When I stuck with them I was like, ‘Oh my gosh, I can go further, higher, longer.’ Then we would increase the resistance, we would increase the band resistance, how they have really light ones and they get heavier, so we could increase those.*” (Ella)
	“*And then once I started building up [strength], I started getting confidence.*” (Lisa)
	“*I had 80% strength when I first saw the physio and did that initial consultation and then probably after three or four months, I probably only had 30 or 40% strength in my shoulder… So, yeah, that was very frustrating after a while because I was like, oh, I am doing these [exercises] properly. And then it probably got to the point where I was kind of like, well, does it really matter if I don’t do it [exercise] tonight, or if I don’t do it tomorrow, and I found [it] really hard to then motivate myself to stick to that same pattern of programming and exercises.*” (Bill)
	“*Even though I’ve seen progress and she’s [clinician] positive and I loved working with her and we had a connection, to me I’m still not back playing pickleball yet. I’m still not back to doing all the things I usually do.*”

### Condition 2: The Exercise Program Should Be Structured and Tailored to the Individual Clinical Presentation

Participants often believed that receiving an exercise program that was tailored to their individual clinical presentation was necessary for their clinical improvement (Tab. 3). Some participants expressed dissatisfaction and skepticism when provided with a seemingly generic exercise program that was not believed to be fit for their purpose. This subsequently affected their engagement and belief in the exercise program, which may have impacted on their clinical outcomes.

Participants frequently reported that an exercise program that was carefully planned and had a logical structure was important to them. Having a plan and structure enabled participants to imagine their possible trajectory over time and gave them something real and tangible they could regularly engage with. This created a feeling of accountability for some participants and may have incentivized continued engagement with the exercise program. When an individualized exercise plan was lacking, this resulted in a feeling of being “left alone.” Based on participants’ responses, an exercise program that is structured and tailored to the individual is an important precondition for exercise to be beneficial for individuals with RCRSP.

### Condition 3: Timely Clinical Progress With Exercise Therapy Matters

The concept of experiencing “timely progress” was an important feature of exercise therapy for most of the participants (Tab. 3). Experiencing timely progress often reinforced to participants that the exercises they were doing were beneficial, thus incentivizing continued adherence to the exercise program. What constituted progress was varied, but most often it included references to increased shoulder strength (eg, lifting more weight or doing more repetitions of the same weight), pain reduction, improved shoulder range of motion (ROM), and the ability to engage with valued recreational pursuits, such as surfing and pickleball. One participant expressed that although they were happy with gaining shoulder ROM and strength, and experiencing less pain, their ultimate goal was to return to pickleball. Progress in psychoemotional states, such as confidence, was also mentioned. Any obvious improvement in physical measures of the shoulder, feelings of confidence using the shoulder, or the ability to perform a certain physical recreational activity were important metrics that participants used to gage their progress. When no discernible progress was experienced, doubt and apathy about the particular exercise program ensued. Thus, if an exercise program failed to elicit a demonstrable change in participant signs and symptoms in a timely fashion, this affected the causal power of the exercise program.

The presence of a strong therapeutic relationship, the provision of a structured and tailored exercise program, and the experience of timely clinical progress were the clinical conditions that promoted positive experiences and outcomes with an exercise program. From the participants’ experiences, we were able to generate 3 causal explanations for the positive clinical outcomes:

### Causal Explanation 1: Shoulder Strength Influences Clinical Outcomes

Most participants reported that increasing shoulder strength or engaging in strengthening exercise was a decisive factor for their positive experience and improvement with exercise therapy (Tab. 4). Some participants reported that they enjoyed the feeling of becoming stronger despite using light weights, while others expressed feeling frustrated when an exercise program was not perceived to be challenging enough. On occasions when a strengthening program was not provided by the treating clinician, this was met with skepticism and doubt. Meanwhile, other participants expressed surprise that their treating clinician encouraged them to try a strengthening–based exercise program and that it was able to produce an improvement in symptoms. There was a belief from some participants that a stretching–based exercise approach or a form of manual therapy was needed for their RCRSP, in part based on past experiences with health care professionals and doing their own research. However, the surprise was often well met by participants, particularly if the treating clinician was able to appropriately explain why they were recommending the said treatment approach.

**Table 4:** Supporting Quotes of Causal Explanations for a Positive Experience With Exercise Therapy

Causal Explanation	Supporting Quotes
Causal explanation 1: Shoulder strength influences clinical outcomes	“*It’s great. I could just feel myself getting stronger even though I’m using two kilo[gram] dumbbells. It’s really light, but I just love it.*” (Claire)
	“*And then the second I started strength training, I felt, yeah, just doing one exercise, every part of my shoulder was engaged. I was feeling that instant connection between the exercise I was doing and a positive improvement in my shoulder.*” (Bill)
	“*And so I felt that that’s what I was doing. I felt those exercise bands or exercises were working muscles I hadn’t used in a long time properly*.” (Ella)
	“*So he did the dry needling and then I just grilled in with questions after. I’m like, what’s now? Am I going to get a strengthening program, and what are your thoughts, what do you think this is? When can I get back to activities, when can I do this*? (Kevin)
	“*I didn’t feel like that was actually building any sort of strength or stability in my shoulder*. *There were exercises that, they were isolating the muscles that they were trying to do, but overall I wasn’t building any sort of strength or resistance to an actual weight that I would be using in real life.”* (Bill)
	“*The strengthening exercises got rid of my pain. It was really bizarre process to me because I thought these sorts of pain things would need stretching, and definitely, I could never lift weights again, and starting to lift weights slowly and doing certain things, like increasing my range in my shoulder with the strength exercises, the pain would just go*.” (Lisa)
	“*I was not expecting him to be taking me to do dumbbell weights. I thought it was different, but I was open to suggestion because I guess he was really confident about it as well. He’s like, ‘You’re weak, this will help’.”* (Claire)
	*“So, even as I progressed strength wise, and I could do heavier things, more complicated things, that same pain would just stay throughout the whole progression [of exercise]… So, that was sort of a bit of, I felt let down by the universe rather than be let down by the exercise, because I was like, “I’m doing this exercise, and it’s worked so, so well for everything else. Why is it not working for this?’”* (Jessica)
	*“So if I had a period where I didn’t go to the gym for three or four weeks or something like that, and then something else happened that was generally when I then instantly start to feel that I was susceptible, if you like, to having more of an underlying issue. But as long as I was maintaining that consistent strength training, yeah, I haven’t had any issues since with my shoulders, which has been really good.”* (Bill)
Causal explanation 2: Exercise therapy influences psychoemotional status	“*But obviously the exercise was important, like I said, for my mental state, to know that I was still physically capable of doing stuff.*” (Max)
	“*Just restoring that faith in my shoulder, I suppose, which is what he got me to eventually do with all of the strength *exercises.” (Lisa)
	*“I went crazy Googling it [bursitis] and it was telling me rest, rest, rest… And I think mentally, if the pain is coming from moving my arm, then I would just stop moving my arm and it wouldn’t give me as much grief, but it was limiting my ability to do things… I hadn’t read anything online about going and getting two and three kilo dumbbells to fix a shoulder injury… It was almost, it felt good to push through the discomfort [when exercising shoulder], knowing that it was for the best, if that makes any sense. So even though it wouldn’t feel the same on both sides, it would feel discomfort and a bit twingey just being told by one person that it’s [exercise] actually not going to hurt me, I was happily able to continue it and know that was for the best… It was more of a mental thing than it was physical.”* (Claire)
	“*So I was very nervous at first [doing shoulder exercises], but I mean, he was watching and walking, doing a 360 and, I guess, checking to see how things were moving. And then when I realized that it wasn’t shooting pain... I think I just kept saying... I kept saying, ‘I can’t believe I’m doing this. I never thought I’d be doing this again’. is what I kept saying to him… ‘Wow, I can’t believe it’. It was almost like it was good. It was having a little mini personal training session because I never thought I’d have that range or the ability to do that again.*” (Claire)
	“*Even though I was doing all the exercises and gaining strength and things, I still didn’t trust myself to do bodyweight exercises…I thought for some reason it [shoulder] would just buckle, but I had exercises building me up to that plank…and then anyway, I eventually got to a plank towards the end, and that’s amazing, and look how far I’ve come.*” (Lisa)
	“*But yeah, I did that [exercises] for a couple of days and it seemed to just take away that fear of using my shoulder and just give me a little bit more confidence that it was sort of working best *again.” (Max)
Causal explanation 3: Exercise therapy has widespread positive health effects	“*It’s a positive. It’s beneficial. Feels good [exercise]… Obviously exercise is a good thing, if done right. It’s good for joints. It’s good for muscle. It’s good for tendons. It’s good for ligaments. It’s good for strength. It’s good for mental health.*” (Luke)
	*“They [exercises] always feel good because it feels like I’m eating a healthy meal or doing a sporting activity. It feels like you’re doing something to get better… So I’ve always put them as this is something that’s good for me, so I get a feeling of achievement at the end of it and I’m doing something good for my body.”* (Kevin)
	*But obviously the exercise was important, like I said, for my mental state, to know that I was still physically capable of doing stuff. But also obviously using it and moving it is probably good for the muscles and keeping things in as best condition, I guess, they could be in.* (Max)

Some participants also expressed that continuing to engage in shoulder strengthening exercise was important for optimizing long-term shoulder health and the prevention of shoulder pain flare ups. One participant reflected on their journey of getting stronger and progressing with their exercise program, but without an associated improvement in pain. This participant felt reassured by their strength gains and simultaneously disappointed that this development of shoulder strength did not eradicate their pain. Taken together, shoulder strength was the dominant causal explanation for a positive experience with exercise therapy according to this cohort of individuals with lived experience of RCRSP.

### Causal Explanation 2: Exercise Therapy Influences PsychoEmotional Status

Participants frequently articulated that the benefits of exercise were in part due its influence on their thoughts and feelings (Tab. 3). In some instances, exercise helped to build or regain trust in their shoulder and also override beliefs that pain during shoulder exercise means it is damaging for their shoulder. There was a tendency for participants to think that rest was the optimal strategy for managing RCRSP, a belief derived mostly from doing their own research. This strategy of rest would sometimes lead to participants overprotecting their shoulder by avoiding basic shoulder movement and exercise. However, engaging in therapeutic exercise that exposed an individual to a movement or exercise that carried an expectation of pain or vulnerability was helpful to change their beliefs about their shoulder pain and capacity. Some participants reported that exercise helped increase confidence in their shoulder, which was sometimes accompanied by reduced fear of using their affected shoulder. Thus, according to the participants in this study, the benefit of exercise for RCRSP goes beyond its biomechanical effects and influences the psychoemotional status of individuals.

### Causal Explanation 3: Exercise Therapy Has Widespread Positive Health Effects

Exercise was often believed to be an intrinsically good activity to engage with due to its varied positive influence on general health (Tab. 3). Exercise was often thought of as a healthy behavior, like eating a nutritious meal, and having multi-system benefits that go beyond the shoulder. This helped the perception of exercise therapy as a beneficial and productive treatment. This may also have helped participants continued engagement with exercise, comfortable in the knowledge that even if exercise didn’t help their shoulder pain specifically or substantially, at least they had done something generally positive for their health. The productive nature of exercise and its perceived capacity to have widespread positive health effects were a common causal explanation for a positive experience with exercise.

## Discussion

This study aimed to gain insights into how individuals with RCRSP believed exercises helped or didn’t help their shoulder pain and identify the clinical conditions that promoted or inhibited this. Whether exercise caused a positive clinical improvement was conditional on certain contextual factors: the presence of a strong therapeutic relationship, the provision of a structured and tailored exercise program, and the experience of timely clinical progress. If these conditions were partially or fully satisfied, exercise therapy consistently produced an improvement in shoulder pain and function, most commonly via the causal explanations: increase in shoulder strength, a positive change in psychoemotional status, and widespread positive influences of exercise on general health.

Most participants in this study expressed that their relationship with their treating clinician was a key part of their experience of engaging with an exercise program for their shoulder pain. A suboptimal or strained therapeutic relationship often led to a poor experience with exercise and the subsequent pursuit of a different health care professional. Conversely, a strong therapeutic relationship was often the foundation for a positive experience with exercise. Thus, the causal power of an exercise program depended on the relationship and interaction between the clinician and individual seeking care, and the better the relationship, typically the better the experience with exercise. The value of a strong therapeutic relationship across diverse non-traumatic musculoskeletal conditions has been discussed in the literature. People with lived experience of patello-femoral pain (PFP),^33^ knee osteoarthritis,^34^ non-specific low back pain,^35^ RCRSP,^36^ and greater trochanteric pain syndrome^37^ consistently express that a trusting, empathetic, and united relationship between themselves and their treating clinician is an integral component of their care. This seems to extrapolate to clinical outcomes too, with evidence reporting a strong therapeutic relationship is associated with improved clinical outcomes.^38^ Crucially, a strong therapeutic relationship may facilitate better adherence to a prescribed exercise program.^39^^,^^40^ Taken together, we conjecture that even the best exercise program might lack causal power in the absence of a strong therapeutic relationship. Thus, we urge clinicians to take time to build this relationship before rushing to provide their expertly crafted exercise program.

Participants who were dissatisfied with a particular exercise approach often lamented that their exercise program was generic and not tailored to their individual goals and needs. A desire for individualized exercise prescription reported in this study is similar to the previous qualitative research evidence for PFP and non-specific low back pain.^33^^,^^41^ The desire for a tailored approach to exercise prescription for RCRSP is supported by empirical data which suggest there is no universal best exercise approach.^42–44^ Therefore, rather than decreeing 1 particular exercise approach as superior, clinicians should endeavor to construct an exercise program in close collaboration with the individual in question and with careful consideration of access to equipment, past experiences with exercise, beliefs about exercise, and their individual goals.

Experiencing timely clinical progress with an exercise program was important for most participants. Most of the time, progress took the form of an improvement in shoulder strength or the experience of less shoulder pain with a particular movement, which aligns with previous qualitative research in the area of non-specific low back pain.^41^ Similar to the work of Littlewood et al,^20^ a lack of timely symptom improvement was met with suspicion and doubt about the benefit of a particular exercise program, which affected motivation and adherence to the exercise program, ultimately leading to poor clinical outcomes. The recovery from RCRSP with exercise is not usually a quick process, and in some cases, it is recommended that 12 months of exercise therapy be trialed prior to escalating treatment to more invasive treatments^45^; the desire for a timely improvement in shoulder symptoms by patients with exercise may prove difficult. Therefore, we encourage clinicians and patients to have open and honest conversations about possible recovery timeframes. Clinicians should consider incentivizing patients to continue with a particular exercise approach by tracking changes in shoulder strength and ROM, using outcome measures that assess ability to perform valued activities (such as the Patient Specific Functional Scale),^46^ and use general patient-reported outcome measures to objectively gauge clinical progress.

The dominant causal explanation of participants in this study for a positive experience with exercise for RCRSP was a perceived improvement in shoulder strength. This finding is concordant with causal explanations proffered by clinical trialists^47^ and practicing physical therapists^5^ for the beneficial effect of exercise for RCRSP. Outside of the shoulder, a similar theme is reported for the effect of exercise for non-specific low back pain^48^ and knee osteoarthritis.^49^ Taken together, the prevailing view of researchers, clinicians, and individuals with lived experience of RCRSP is that “getting stronger” is the key mechanism underpinning the benefits of exercise therapy for RCRSP. This is despite there being no empirical support that shoulder strength is a mediator of outcomes for individuals with RCRSP.^50^ It remains biologically plausible that shoulder strength is causally linked to positive clinical outcomes in people with RCRSP, based on evidence which shows that quadriceps strength mediates improvements in pain and physical function in individuals with knee osteoarthritis.^51^ Therefore, the biological plausibility and consistent reports from different stakeholders regarding the importance of shoulder strength means that it is a casual explanation researchers and clinicians should take seriously.^52^^,^^53^ Another advantage of using shoulder strength as a useful heuristic to causally explain the effect of exercise for RCRSP is that it is a cogent word that individuals with varying degrees of health literacy can understand. Clinicians should cautiously consider using shoulder strength as a valid and comprehensible causal explanation for the beneficial effect of exercise. Further research is required to strengthen this proposal.

Various psychoemotional states were expressed by participants as being influenced by exercise therapy, including increased confidence and belief in their shoulders, reduced fear of shoulder movement, and reassurance that their painful shoulder was still able to perform basic activities. Kinesiophobia and pain self-efficacy are associated with clinical outcomes in people with shoulder pain^54–56^; however, it is uncertain to what extent exercise can modify these constructs. Research on osteoarthritis has shown self-efficacy to be a mediator of clinical outcomes^57^ and this could well be the case for RCRSP.^58^ There is also low-quality evidence that exercise training can effectively reduce fear avoidance beliefs in a predominately spinal pain cohort (cervical and lumbar spine).^59^ Some participants alluded to experiencing a psychological phenomenon with exercise therapy that resembles expectancy violation. Expectancy violation^60^ is a concept embedded within exposure therapy, which has been a mainstay of treatment for anxiety disorders for decades.^61^ When applied to musculoskeletal pain, expectancy violation represents the discrepancy between expected and experienced pain during a specific movement or task. It is theorized that a mismatch between expectation and experience is critical for new learning^61^^,^^62^ and the updating of possible unhelpful understandings about pain. Some participants in this study expressed surprise that they could lift a dumbbell weight without pain or that they could work up to doing bodyweight exercise despite initially not trusting their shoulder. This surprise or “expectancy violation” may have contributed to enhanced feelings of confidence in their shoulders and provided reassurance and motivation that they were on the right pathway to recovery. Based on the experiences of the participants in this study and also the current evidence base, we contend that various psychoemotional states underpin a positive experience with exercise for RCRSP, and clinicians should consider building this into their education.

Participants in this study often acknowledged that exercise can positively influence multiple bodily systems. This sentiment agrees with an abundance of evidence reporting the positive association between exercise and general health and wellbeing.^63–68^ Exercise was often recognized as a fruitful treatment to engage with because of its healthy behavior connotations, which may have led to perceived improvement in shoulder symptoms. Taken together, causal explanations for the benefit of exercise for RCRSP could involve references to the whole-body effects of exercise.

It is important to clarify that the causal mechanisms of exercise that led to a positive clinical outcome in this cohort of individuals with RCRSP were only triggered in the presence of an appropriate clinical context (Figure). That exercise has causal powers which depend on its manifestation partners (individual with RCRSP and clinician) is a novel theory when specifically applied to exercise and RCRSP but fits comfortably within established critical realism philosophy^69^ and dispositionalist theories of causation.^70–72^ If we take this theory to its logical conclusion, there probably is no magical dose or type of exercise that will universally help all individuals with RCRSP—because every individual will present with their own set of personal dispositions which may or may not respond to a particular exercise program or the prescribing clinician. Any exercise program for RCRSP has the potential to help 1 individual, harm another, or have no effect.^73^ Person-centered health care acknowledges the medical uniqueness of an individual, and it makes pragmatic and philosophical sense to use exercise therapy with this theory in mind.^71^

**Figure:**
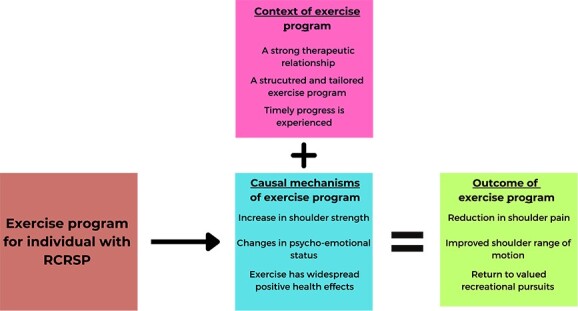
The relationship between Intervention-Context-Mechanism-Outcome for individuals with rotator cuff–related shoulder pain (RCRSP).

## Limitations

There are methodological considerations to this study which warrant attention. The sample size of 11 participants may be considered small from a quantitative research standpoint. However, it is important to emphasize that this is an original qualitative research investigation, and the sample size was derived by achieving theoretical sufficiency. The lead researcher was a novice interviewer but is an experienced musculoskeletal clinician well versed in communicating with people with shoulder pain, who also undertook training in qualitative research interviewing prior to commencing the study. All interviews were conducted via online video conferencing software, which could have affected researcher-participant rapport, although evidence does support the use of this method of interviewing.^28^ Furthermore, data were predominately collected from people from economically developed countries whose first language was mostly English. It is important to acknowledge and respect that navigating pain, health care, and exercise for a particular musculoskeletal pain condition is influenced by cultural aspects and thus the findings of this study may not be transferable to other sociocultural contexts. Future research efforts might consider replicating the research methods used in this study and applying to a cohort of individuals from economically developing countries.

## Conclusion

In summary, the most common causal explanations for a positive experience with exercise in individuals with RCRSP were an increase in shoulder strength, changes to psychoemotional status, and the belief that exercise has widespread positive health effects. However, these causal mechanisms were only activated in the clinical context of a strong therapeutic relationship, the provision of a structured and tailored exercise program, and the experience of timely clinical progress. Clinicians should consider not just the exercise parameters of an exercise program but also the clinical context in which the exercises are prescribed.

## Data Availability

The data that support the findings of this study are available from the corresponding author, [JKP], upon reasonable request.
